# Mercury accumulation over the Holocene revealed from a Greenlandic ice core

**DOI:** 10.1126/sciadv.aea0517

**Published:** 2026-05-15

**Authors:** Zhiyuan Gao, Richard Oliveira, Aryeh Feinberg, Delia Segato, Alfonso Saiz-Lopez, Debbie Armstrong, Andrea Spolaor, Helle Astrid Kjær, Jørgen Peder Steffensen, Carlo Barbante, Dorthe Dahl-Jensen, Feiyue Wang

**Affiliations:** ^1^ Centre for Earth Observation Science, and Department of Environment and Geography, University of Manitoba, Winnipeg, MB R3T 2N2, Canada.; ^2^ Department of Atmospheric Chemistry and Climate, Institute of Physical Chemistry Blas Cabrera, CSIC, Madrid 28006, Spain.; ^3^ Institute of Polar Sciences, CNR-ISP, Campus Scientifico Via Torino, Venice-Mestre, 30172, Italy.; ^4^ Department of Environmental Sciences, Informatics and Statistics, University Ca’ Foscari of Venice, Venice-Mestre, 30172, Italy.; ^5^ Centre for Ice and Climate, Physics of Ice, Climate and Earth, Niels Bohr Institute, University of Copenhagen, Copenhagen DK-2200, Denmark.

## Abstract

Mercury is a contaminant of global health concern, but anthropogenic impact on preindustrial mercury cycling remains poorly constrained. Here, we report a high-resolution Greenlandic ice core record of the mercury concentration and accumulation flux over the entire Holocene. We show that the Greenlandic mercury accumulation was shaped by volcanic eruptions, climate excursions, and anthropogenic activity in the past millennia. Mercury accumulation has increased remarkably since the 13th century and further intensified in the past two centuries, which closely mirrors estimated anthropogenic emissions. However, anthropogenic impact was evident long before that, as mercury accumulation entered multiple periods of continuous increase for almost two millennia following early anthropogenic mercury uses. Our result suggests that human activity might have started to impinge on Greenlandic mercury cycling earlier than previously thought and that assessing all-time anthropogenic mercury emissions needs to account for a longer period than the current estimates.

## Teaser

A Greenlandic ice core mercury record suggests human impact on mercury cycling might have started earlier than previously thought.

## INTRODUCTION

Mercury (Hg) is a naturally occurring element, and its global cycling has been largely influenced by human activity (*1*). In 2017, the Minamata Convention on Mercury entered into force, aiming to protect the ecosystem and human health from Hg pollution by reducing anthropogenic Hg uses (*2*). Our ability to assess the effectiveness of the Convention and project ecosystem recovery from Hg pollution is challenged by uncertainties associated with legacy anthropogenic Hg releases throughout human history (*3*, *4*). It is well known that cinnabar (HgS) had been mined in small quantities as vermillion for more than 5000 years in Europe (*5*) and for more than 3000 years in Asia (*6*) and South America (*7*). Anthropogenic uses of Hg increased markedly since the 16th century for gold and silver mining (*8*–*11*), which were further intensified since the mid-19th century due to the Gold Rush and large-scale industrial productions (*3*, *4*, *12*, *13*). However, the onset and cumulative magnitude of preindustrial anthropogenic perturbation on global Hg cycling, especially in remote polar regions, remain largely unconstrained.

Glacier ice cores may provide the key to addressing these questions as legacy Hg emissions to the atmosphere could be preserved through direct atmosphere-snow-ice interactions. Once emitted to the atmosphere primarily as gaseous elemental Hg [Hg(0)], Hg is readily redistributed from the source region to remote polar locations with an atmospheric lifetime of 4 to 8 months (*14*, *15*), during which some of the Hg can be oxidized to Hg(II). In polar regions, Hg enters the surface cryosphere via air-snow exchange [for Hg(0); (*16*)] and wet and dry deposition [for Hg(II); (*17*)]. The retention of Hg in the snowpack is subject to dynamic postdepositional processes resulting in rapid photoreduction of Hg(II) and re-emissions of Hg(0) (*18*, *19*), leading to seasonal variations in the snowpack. Once the remaining Hg is buried deeper into the snowpack, it is preserved with smoothed seasonal variations (*20*). Eventually, Hg in the snowpack is registered as ice core Hg accumulation and can be used as an indicator for atmospheric Hg deposition following modifications from postdepositional processes (*21*–*23*).

Alpine glacier ice cores retrieved from Asia and North America and polar firn air measurements from Greenland have established high-resolution continuous Hg records from the present day back to the early 15th century (*21*–*24*), corresponding to the period with the most intense anthropogenic Hg uses. Deep ice cores from the Antarctic and Greenlandic Ice Sheets have also been used to examine natural Hg variabilities during glacial periods before the Holocene (*25*, *26*). Yet, no such ice core Hg record has covered the entire Holocene at high temporal resolution.

**Fig. 1.**
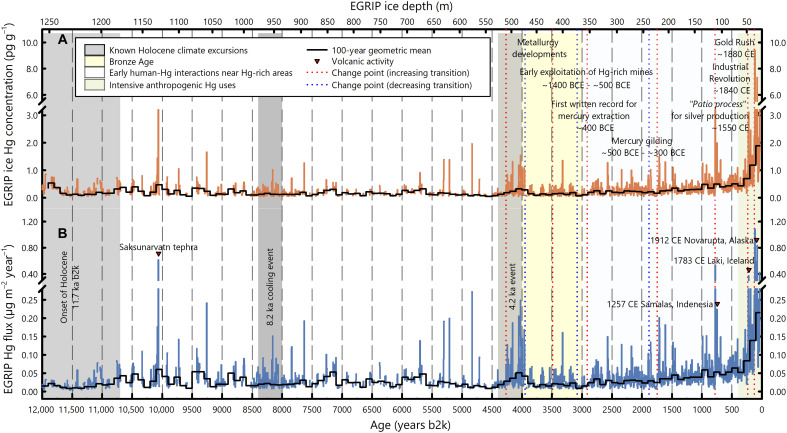
The mercury record over the Holocene as measured from the EGRIP ice core. (**A**) Mercury concentrations. (**B**) Mercury accumulation fluxes. Known periods of Holocene climate excursions are marked in gray shades, including the onset of the Holocene, the 8.2-ka event, and the 4.2-ka event. Anthropogenic activity potentially affecting the Greenlandic Hg deposition is identified in three periods: the Bronze Age, known early human-Hg interactions near Hg-rich areas, and the period of intensive anthropogenic Hg uses in gold and silver mining and industrial production. The solid step curve represents the geometric mean for each century. Red and blue dotted lines represent identified abrupt change points in the recent 4500-year continuous Hg measurements.

With the recently retrieved deep glacier ice core from the East Greenland Ice Core Project (EGRIP) drilled on the Northeast Greenland Ice Stream (fig. S1), we present here a Holocene record of ice core Hg concentrations and accumulation fluxes at multiannual resolution (Fig. 1). The high-resolution record allows us to examine how early human-Hg interaction and climate might have affected preindustrial Greenlandic Hg accumulation throughout the Holocene.

## RESULTS

Ice core samples from the uppermost EGRIP ice core sections (depth range: 14 to 1254 m) were analyzed for the total Hg concentration, corresponding to a time period from 12.013 thousand years (ka) before the year 2000 (b2k) to 34 years b2k (i.e., 1966 CE) in the GICC05 timescale (*27*); the age-depth profile of the ice core and variations in the temporal resolution are provided in fig. S2. The temporal resolution ranges from 4 to 6 years for the most recent period of 34 years to ~8.0 ka b2k to 5 to 10 years for the period older than ~8.0 ka b2k. The Hg record includes continuous measurements (i.e., every 55-cm ice core section, which is referred to as one EGRIP bag) for the recent 4500 years and the two periods covering known Holocene climate excursions [11.0 to 12.0 ka b2k; 7.5 to 8.5 ka b2k; (*28*)]. For the rest of the Holocene, 10 consecutive EGRIP sample bags (i.e., 5.5 m of 40 to 60 years) were analyzed for every 30 bags (i.e., 16.5 m of 120 to 180 years).

Throughout the Holocene, the ice Hg concentration ranged from below the detection limit (DL; 0.05 pg g^−1^) to 9.4 pg g^−1^. As the measured Hg concentrations and calculated Hg fluxes generally follow a log-normal distribution, the average Hg concentrations and fluxes are presented hereafter as the geometric mean along with the 95% confidence interval (CI) in the brackets. For the recent millennium, the Hg concentration ranged from 0.07 to 9.4 pg g^−1^ with a geometric mean of 0.62 (0.55 to 0.68) pg g^−1^ (*n* = 223), which is at the lower end of the range reported for glacier ice cores corresponding to the similar time period retrieved from Asia (*22*, *23*), North America (*21*, *29*, *30*), and the Canadian Arctic and Greenland (*31*, *32*) (fig. S3 and table S1). However, ice core Hg concentrations reported here for the early Holocene are much lower than those reported for the glacial periods in the Antarctic (*26*).

Based on the modeled EGRIP snow accumulation record (*33*), the calculated Hg flux (see Materials and Methods) ranged from 0.002 to 1.08 μg m^−2^ year^−1^. The Hg flux is found to correlate strongly with the ice core Hg concentration (*r* = 0.99, *P* < 0.01) and weakly with the snow accumulation rate (*r* = −0.11, *P* < 0.01).

Since the onset of the Holocene, the Hg concentration and accumulation flux oscillated at generally low levels with no remarkable changes until the late Holocene when both increased considerably. Superimposed on the long-term trend, there were sporadic and sharp spikes that lasted for less than a decade each throughout the Holocene.

To identify potential abrupt changes during the late Holocene, a change point analysis was performed with continuous measurements of log-transformed Hg concentration and accumulation flux during the recent 4500 years (see Materials and Methods). In total, 10 change points were identified consistently (Fig. 1 and table S2), with 7 of them corresponding to an increase (126, 235, 781, 1746, 2913, 3490, and 4266 years b2k) and 3 to a decrease (1882, 3081, and 3949 years b2k). These change points delineate periods of Hg oscillations during the late Holocene. For instance, beginning with an increase at 4266 years b2k and ending with a decrease at 3949 years b2k, the Hg accumulation flux elevated to 0.044 (0.036 to 0.055) μg m^−2^ year^−1^ (*n* = 60), which was still relatively low compared to the entire Holocene. Since 1746 years b2k (i.e., 254 CE), there have been three increase points with no intervening decrease, suggesting a period with consistently accelerating increases in the Hg concentration and accumulation flux. This is further confirmed by a trend analysis (fig. S4) and distribution histograms (fig. S5). The most recent increasing period started from 126 years b2k (i.e., 1874 CE), with the Hg accumulation flux reaching 0.235 (0.187 to 0.296) μg m^−2^ year^−1^ (*n* = 30).

## DISCUSSION

The EGRIP ice core offers a natural archive to explore preindustrial Holocene variations in atmospheric Hg, complementing with earlier insights derived from sediment and peat bog archives (*34*–*36*). Previous ice core studies with the FLEXPART atmospheric transport and deposition modeling have shown that the Greenlandic ice core record of lead (Pb), which is transported primarily as particles (*37*), tracks emissions from North America and western Europe (*38*, *39*). As Hg is both volatile [Hg(0)] and particle active [Hg(II)], variations in the EGRIP Hg accumulation as shown in Fig. 1 are expected to track Hg emissions from most of the Northern Hemisphere, including North America, Europe, and possibly Asia. It should be noted that ice core Hg variations are also subject to changes in surface postdepositional processes, which remain largely unconstrained due to our limited knowledge of paleoclimate conditions. For the discussions below, we assume that the relatively stable ultraviolet irradiance (*40*) and the relatively constant snow accumulation rates (*33*), especially during the late Holocene, imposed negligible changes in Hg postdepositional processes. Accordingly, the data interpretation will focus mainly on ice core Hg accumulation as an indication for atmospheric Hg deposition.

### Short-lived natural events

Throughout the Holocene, sharp spikes in the Hg concentration and accumulation flux were mainly associated with short-lived natural events mobilizing geogenic Hg into the atmosphere, among which volcanism has been recognized as a dominant process (*41*). The registration of Hg flux spikes in alignment with volcanic activities has been found in some lake sediment and peat cores in proximity to eruptions (*42*–*44*), but identification of volcanic Hg in glacier ice cores is less straightforward due to greater uncertainties associated with the location and type of the eruption and the meteorological conditions at the time of eruption (*22*, *23*, *30*, *41*).

Nevertheless, the highly resolved EGRIP Hg record allows a preliminary allocation of sharp Hg flux spikes to known large volcanic eruptions (*45*) on a timescale of less than a decade. For instance, elevated Hg fluxes above the background level can be attributed to explosive eruptions in the Northern Hemisphere (e.g., the 1912 Novarupta eruption in Alaska, the 1257 Samalas eruption in Indonesia) and nearby Icelandic eruptions (e.g., the 1783 Laki eruption in Iceland) (Fig. 1).

Sharp Hg flux spikes have also been found near known tephra layers. For example, the largest Hg flux (0.61 μg m^−2^ year^−1^) in the early Holocene recorded at 10.059 ka b2k can be attributed to the influence from the Saksunarvatn tephra (*46*) (Fig. 1).

### Holocene climate excursions

The Hg accumulation was also sensitive to longer period changes associated with climate excursions, such as changes in dust deposition (*26*), sea ice conditions, and atmospheric bromine (Br) chemistry (*25*). Compared to the Dansgaard-Oeschger events during the Last Glacial Period, the Holocene climate varied at a milder extent (*47*). Known Holocene climate events include the onset of the Holocene and the 8.2-ka cooling event that were recorded prominently in the Greenlandic ice cores (*47*, *48*) and the 4.2-ka event that was evident elsewhere (*28*) but not clearly recorded in the Greenlandic ice core.

**Fig. 2.**
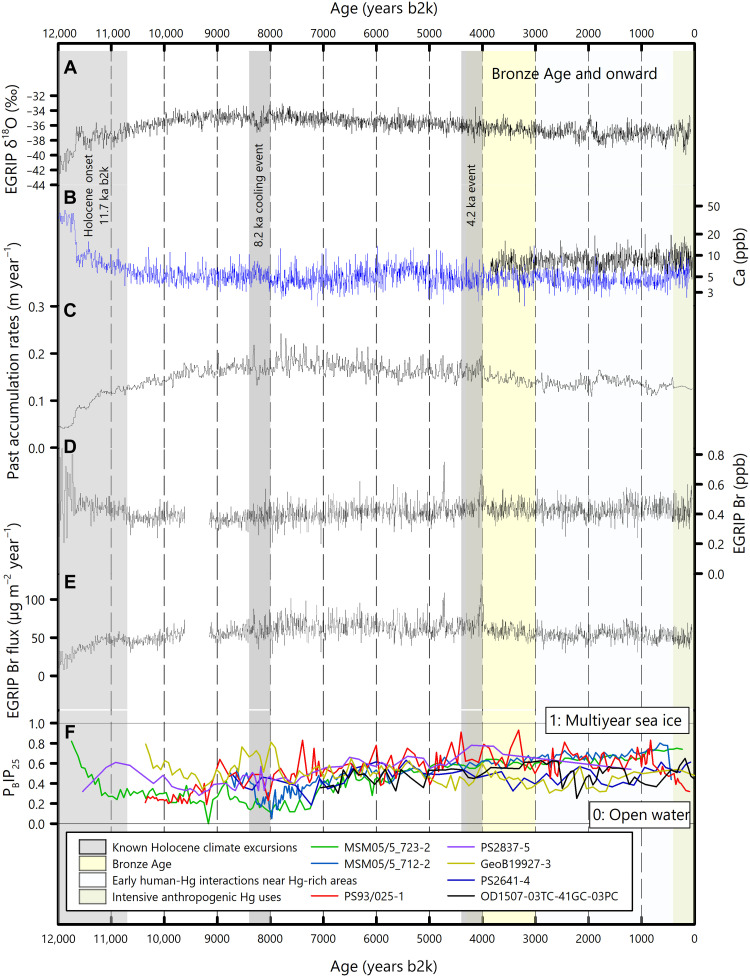
Potential environmental proxies influencing EGRIP mercury accumulation over the Holocene. (**A**) δ^18^O values measured from the EGRIP ice core (*57*). (**B**) Calcium (Ca) concentrations over the entire Holocene from the GISP2 ice core (in blue) (*47*) and during the recent 3800 years from the EGRIP core (in black) (*60*). (**C**) Snow accumulation rates determined from the EGRIP core (*33*). (**D**) Bromine (Br) concentrations measured in the EGRIP core. (**E**) Calculated Br fluxes at EGRIP. (**F**) P_B_IP_25_ measurements in marine sediment cores retrieved from the east [MSM05/5_732-2 (*55*), MSM05/5_712-2 and PS2641-4 (*56*), PS2837-5 (*51*, *52*), and PS93/025-1 (*54*)] and west [GeoB19927-3 (*50*) and OD1507-03TC-41GC-03PC (*53*)] coasts of Greenand.

The transition from the cold Younger Dryas into the warm Holocene was accompanied with a slight increase in the EGRIP Hg flux until ~9.0 ka b2k; a similar pattern was also evident in elevated Hg concentrations found in sediment cores from North America (*34*, *49*). Contemporary with this increase were a period of increasing snow accumulation rates (*33*), an extended open water period around Greenland as indicated by a decreasing P_B_IP_25_ index (a brassicasterol-based proxy of sea ice diatoms) (*50*–*56*), and greater atmospheric Br concentrations inferred from an increasing EGRIP Br flux (Fig. 2). As a result of changing regional environmental proxies, higher oceanic Hg(0) evasion and intensified snow deposition of Hg(II) resulted in an increase in the Greenlandic Hg accumulation, corroborating that changes under oceanic and sea ice conditions were primary drivers for Hg variabilities during the early Holocene (*25*). During the abrupt cooling period of 8.4 to 8.0 ka b2k (i.e., the 8.2-ka event), the Hg flux remained low at 0.020 (0.017 to 0.024) μg m^−2^ year^−1^ (*n* = 67).

A more pronounced increase in the Hg flux occurred during the 4.2-ka event (4266 to 3949 years b2k), when the Hg flux increased by 214% to 0.044 (0.036 to 0.055) μg m^−2^ years^−1^ (*n* = 60), from 0.014 (0.012 to 0.017) μg m^−2^ year^−1^ (*n* = 77) during 5000 to 4266 years b2k. The increase was accompanied with two notable changes in temperature and the Br concentration. During the 4.2-ka Hg flux increase, a warm anomaly superimposed on the gradual cooling trend was found in EGRIP δ^18^O values that increased from −38.4 to −34.7‰ over ~100 years (*57*) (Fig. 2A), corresponding to a temperature increase of ~5.4 K based on the empirical δ^18^O thermometer (~0.67‰ K^−1^) (*58*, *59*). During the same period, the Br flux almost doubled from 63.4 ± 11.8 μg m^−2^ year^−1^ (mean ± 1 SD) during 5.0 to 4.5 ka b2k to 119.5 μg m^−2^ year^−1^ around 4.0 ka b2k (Fig. 2E). The increased atmospheric Br concentrations would have caused intensified Hg oxidation and deposition to the ice sheet where the Hg accumulation was also favored by slightly increased snow accumulation rate during the 4.2-ka event (Fig. 2C).

On the other hand, dust deposition remained low during the 4.2-ka event, as indicated by the calcium (Ca) concentration in a nearby deep ice core [Greenland Ice Sheet Project 2 (GISP2)] (*47*) that varied similarly to that in the EGRIP ice core (*60*) (Fig. 2B). Variations in ice core Ca concentrations have been widely used to quantify mineral dust aerosols (*61*) and identify sources of wind-blown dust particles (*62*, *63*). Although Ca concentrations fluctuated within the Holocene natural variability during the 4.2-ka Hg flux increase, its overall magnitude of variation was much lower than that during the transition from the Younger Dryas into the Holocene when dust-bound Hg was thought to have played a minor role in causing the Hg flux increase (*25*). Given the overall low Ca concentrations, dust-bound Hg was not expected to play a major role in the 4.2-ka Hg flux increase.

In addition, during this period, sea ice conditions around Greenland either remained relatively stable or showed a slightly accreting pattern toward a multiyear sea ice coverage as indicated by the P_B_IP_25_ index (*50*–*56*) (Fig. 2F), which would have impeded open water Hg evasion and caused a Hg flux decrease. Another possible mechanism responsible for the 4.2-ka Hg flux increase is the Saharan desertification as the large-scale climate-induced deforestation in North Africa might have elevated atmospheric Hg concentrations (*64*). However, the ecosystem succession in North Africa was gradual over the Holocene (*65*), not aligning with the rate of the 4.2-ka Hg flux increase (see text S2 in the Supplementary Materials for discussion on the long-term trend of ice core proxies).

To resolve the potential contributions from Br and temperature during the 4.2-ka Hg flux increase, a box model was used to simulate atmospheric chemistry influencing Hg deposition (see Materials and Methods). The result shows that with an 88% increase in the Br flux alongside a 5 K increase in temperature, it would have only resulted in an ~53% increase in the Hg flux (range: 24 to 92% as the 5 to 95% percentiles; fig. S6), accounting for only a small fraction of the observed 214% Hg flux increase during this period (Fig. 1).

The 4.2-ka Hg flux increase thus cannot be solely attributed to known climate-driven factors. Either there were other uncharacterized climate-driven processes at play (e.g., variations in the snow Hg re-emission process) or it could have signaled the impact of early human activity as detailed below.

### Anthropogenic activity

Because the 4.2-ka Hg flux increase cannot be solely explained by known climate-driven factors, we postulate that it could be attributed, at least in part, to early human activity as the timing coincided with several major milestones in human history, notably the civilization transition from the Neolithic to Bronze Age. The advent of the Bronze Age resulted in early metallurgical development and spreading of agriculture in Eurasia starting around 2300 to 2200 BCE (*66*, *67*). At that time, Hg probably remained unknown to the civilization, but the pursuit of copper and tin ores could have released Hg from impurities in sulfide deposits (*68*, *69*) by thermal decomposition during fire setting (*70*), smelting, and bronze casting. In addition, deforestation due to spreading of agriculture and land cultivation during the expansion of the Bronze Age could have also contributed to unintentional Hg emissions (*64*).

A localized Hg flux increase during the Bronze Age has been observed in sediment cores from Europe and the Mediterranean Sea (*71*–*74*); if our postulation holds, the EGRIP Hg record would suggest that the Bronze Age impact on Hg cycling had reached as far as Greenland via atmospheric transport. However, we caution that further evidence is needed to support anthropogenic impact on Hemispheric-scale Hg cycling in the Bronze Age, because it predated the known history of early Hg mining and uses by ~1000 years. A similar dilemma exists in the methane budget during the late Holocene, as unexpected methane increases recorded in ice cores have to be explained by both anthropogenic and natural sources (*75*). The potential anthropogenic perturbation on the methane budget around 5000 years ago was considered the start of the anthropogenic greenhouse era with early land use for irrigation (*76*), within which lies the observed 4.2-ka Hg flux increase.

During the late Holocene, sediment and peat bog archives have suggested local-scale Hg increases near Hg-rich mines due to the start of Hg exploitation (*6*, *7*, *77*). In comparison, the EGRIP ice core Hg record showed a millennial-scale increasing trend since ~1000 BCE (change point at 2913 years b2k), suggesting that Hg(0) emissions from early Hg uses may have been transported via atmospheric circulation and delivered to Greenland. In addition to early mining activity, the period also marked the beginning of cinnabar smelting around 400 BCE (*78*), where the heating process could have readily released gaseous Hg(0) to the atmosphere. Later on, during 500 to 300 BCE, the invention of Hg amalgamation in artifact decoration as Hg gilding made the element popular in Europe and China (*79*, *80*). Since 254 CE (change point at 1746 years b2k), the Greenlandic Hg record showed multiple periods of increasing accumulation, generally following known early anthropogenic Hg uses near Hg-rich areas. The observed millennial-scale Hg flux increase in the EGRIP ice core complements multiple sedimentary Hg records in South America, Europe, and Asia although with different magnitudes and timelines (*35*, *72*, *81*). The discrepancy could be due to the distinctive regional development patterns and the sensitivity to atmospheric Hg deposition among various natural archives. Our results thus suggest that anthropogenic activity has impinged on Greenlandic Hg accumulation for more than two millennia since 254 CE and potentially earlier following the emergence of early anthropogenic Hg uses. However, we caution that anthropogenic contribution during this early period cannot be properly quantified on the basis of the ice core Hg concentrations; other information such as ice core Hg isotopic composition would be needed.

Because of uncertainties under paleoclimate conditions, it is difficult to estimate a single value for natural Holocene Hg background from the EGRIP ice core record. For the purpose of data comparison, the period during 10,000 to 1746 years b2k is considered the baseline because it excluded the immediate transition from the Last Glacial Period into the Holocene and predated large-scale anthropogenic Hg uses (see text S1 in the Supplementary Materials for discussion on the natural baseline). This approach yielded a Holocene Hg accumulation baseline of 0.022 (0.021 to 0.023) μg m^−2^ year^−1^ (*n* = 1008). In comparison, the post–254 CE period showed a Hg flux of 0.056 (0.052 to 0.060) μg m^−2^ year^−1^ (*n* = 378), corresponding to an ~1.5 times increase.

**Fig. 3.**
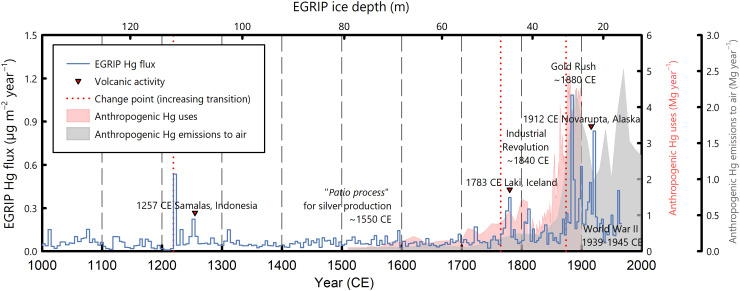
Comparison of the EGRIP Hg accumulation flux at the multiannual resolution since 1000 CE with the estimates of anthropogenic mercury uses and emissions over the most recent 500 years. Red dotted lines represent identified abrupt change points in the continuous Hg measurements. The data for anthropogenic Hg uses (in red) are obtained from Guerrero and Schneider (*4*) and those for anthropogenic Hg emissions to the air (in gray) from Streets *et al.* (*3*), both of which were based on the Northern Hemisphere, including Europe, North America, and Asia.

The considerable Hg flux increase since the 13th century (change point at 781 years b2k), especially during the industrial era, featured the period with intensive anthropogenic Hg uses (Fig. 1; for zoomed-in variations, see Fig. 3). This period encompassed the use of Hg for silver production in the colonial New World since the 1600s, which is arguably the most extensive use of Hg in human history (*8*, *9*). However, Hg used in the “patio process” for silver refining did not result in the highest Greenlandic Hg accumulation in the Holocene. This is because most of the Hg loss from the “patio process” was chemically sequestered locally through the formation of calomel, limiting its Hg(0) emissions to the atmosphere (*82*, *83*). In contrast, a more rapid increase in the Hg flux was observed later in the late-19th century during the Gold Rush in North America, when Hg used for gold extraction was readily lost to the atmosphere (*82*). The most recent Hg flux increase identified in the EGRIP Hg record is the period since 1874 CE (change point at 126 years b2k), which agrees very well with other natural archives from different locations (*84*, *85*). A slight decrease in the Hg flux occurred around the 1940s due to reduced industrial production during the Second World War (Fig. 3). Since then, the flux gradually rebounded with a rapid recovery of industrial production and continued through the 1960s, which was the most recent data point recorded in the EGRIP ice core. The subsequent decrease in the Hg accumulation due to reduced anthropogenic Hg emissions, which is commonly observed from sediment records, postdated the latest EGRIP ice sample. These observed trends of the Greenlandic Hg accumulation flux, especially during the past 200 years, agree very well both in timing and magnitude with the estimated anthropogenic Hg emissions (Fig. 3), affirming that emission-driven atmospheric Hg variabilities were well preserved in the EGRIP ice core. Thus, the 13th century may be considered the beginning of considerable human perturbation on Greenlandic Hg accumulation with the recent several centuries driven by intensified anthropogenic emissions. Since then, the Hg flux reached 0.082 (0.074 to 0.092) μg m^−2^ year^−1^ (*n* = 178), which is 2.7 times higher than the pre–254 CE Holocene baseline. For the industrial era since 1840, the Hg accumulation rate was as high as 0.187 (0.150 to 0.233) μg m^−2^ year^−1^ (*n* = 41), representing a 7.4 times increase relative to the pre–254 CE Holocene baseline.

In summary, the EGRIP ice core Hg record shows that Greenlandic Hg accumulation over the Holocene was shaped by a combination of volcanic eruptions, climate excursions, and anthropogenic activity. Our results suggest that anthropogenic activity has perturbed Greenlandic Hg accumulation for more than two millennia (1.5 times increase over the Holocene baseline), with considerable accumulation increases since the 13th century (2.7 times increase over the Holocene baseline), which further intensified since 1840 CE (7.4 times increase over the Holocene baseline). Our results further caution that the currently used emission inventories that are primarily weighted over the most recent five centuries might have underestimated considerably the all-time anthropogenic Hg emissions. Further studies are needed to revisit the complex human-Hg relation that may have started as early as in the Bronze Age, and to better quantify the all-time anthropogenic contribution to Hg deposition (e.g., through high-resolution ice core Hg isotopic measurements), which could have major implications for improving global Hg transport modeling and for assessing the effectiveness of the Minamata Convention.

## MATERIALS AND METHODS

### Ice core collection

As a collaborative international effort, the EGRIP program started in 2016 to retrieve a deep glacier ice core on the Northeast Greenland Ice Stream. The drilling site is located at 75°38.05′N and 36°00.22′W at 2708 m above sea level and drilling reached the bedrock in 2023 with a total ice depth of 2670 m. Ice core chronology, parameters such as aerosols and δ^18^O, and upstream ice-flow modeling have been reported for various segments (*27*, *33*, *60*, *86*), supporting the interpretation of Hg concentrations and fluxes reported in this work. To cover the entire Holocene range, Greenland ice core chronology GICC05 was used for this study (*27*). We acknowledge a small discrepancy in chronology between GICC05 and the latest published GICC21, estimated to be 13 years at 3835 years ago (*86*).

All EGRIP ice core samples were archived in 55-cm sections (“EGRIP sample bag”) at the University of Copenhagen. Subsampling for Hg was done at a walk-in freezer (−18°C) by cutting the ice with a band saw equipped with a stainless steel blade, following the “clean-hands-and-dirty-hands” protocol (*87*). To cover the entire depth of an EGRIP bag (“bag-mean sample”), a thin ice stick (1.3-cm wide, 55-cm long, ~75 g) was cut from the side of the core sections. An extensive and thorough ice cleaning process was used to ensure the data quality when measuring sub-parts-per-trillion levels of Hg. The ice stick was cleaned by scraping off, using an acid-cleaned ceramic knife, all exterior surfaces that might have been in contact with the drilling fluid during retrieval, bags during storage, and the band saw during cutting to eliminate cross-contamination. The cleaned samples (~50 g) were stored in new polypropylene tubes (Falcon) that were randomly tested to contain Hg concentrations below the DL (see below). For ice core samples below the depth of 205 m, the scraping was done under a portable Class-10 laminator flow environment in the same walk-in freezer.

Three types of blanks (Milli-Q water stored in the same type of sampling tubes) were also collected to ensure the quality of the sample cutting process, including travel blanks that were not opened during ice processing, open blanks that were opened briefly in the walk-in freezer, and open blanks that were opened briefly under the laminator flow.

All the ice samples and blanks were stored in airtight coolers and transported to the Class-100 Ultra-Clean Trace Elements Laboratory (UCTEL) at the University of Manitoba (Canada). The samples remained frozen or partially frozen upon arrival.

### Mercury analysis

Once the ice melted completely in the dark, an aliquot of the meltwater (~30 ml) was preserved with Hg-free HCl (CMOS grade, JT Baker) to 0.5% (v/v). The meltwater was analyzed for total Hg following the modified EPA 1631 method on a Tekran 2600 cold vapor atomic fluorescence spectrophotometer (CVAFS) (*88*). Briefly, samples were treated with BrCl (JT Baker) oxidation for at least 8 hours before analysis, followed by SnCl_2_ (Fisher Chemical) reduction. The method thus measures all BrCl-oxidizable Hg (“total Hg”) in the solution. To ensure the accuracy and precision of Hg analysis, a certified reference material, coastal seawater BCR-579 (Institute for Reference Materials and Measurements), was measured repeatedly during analysis, yielding values of 1.97 ± 0.17 pg g^−1^ (*n* = 61) that agreed well with the certified value of 1.90 ± 0.50 pg g^−1^. The method DL was calculated on the basis of repeated measurements on a low Hg solution (0.5 pg g^−1^), and was found to be 0.05 pg g^−1^ (*n* = 26). Mercury concentrations were below the DL in the travel blanks (*n* = 15), 0.11 ± 0.08 pg g^−1^ (mean ± 1 SD; *n* = 21) in the freezer open blanks, and 0.06 ± 0.04 pg g^−1^ (mean ± 1 SD; *n* = 28) in the open blanks under the Class-10 laminator flow.

Among all 1538 measured ice core samples analyzed covering the Holocene, 52 were found to have Hg concentrations below the DL and were assigned a value equal to half of the DL for the Hg flux calculation. It should be noted that Segato *et al.* (*25*) reported higher Hg concentrations in the same EGRIP ice core for the overlapping period (12.0 to 9.0 ka b2k) using meltwater samples collected during the continuous flow analysis (CFA).

### Bromine analysis

Bromine (Br) analysis was done on EGRIP ice core samples collected from the CFA system at the University of Bern. The samples were immediately frozen at −20°C and transported to Ca’ Foscari University of Venice (Italy) for analysis. Total Br concentrations were determined on a Thermo Fisher Scientific iCAP inductively coupled plasma mass spectrometer. Bromide standard solutions were prepared at 0.01, 0.05, 0.1, 0.25, 0.5, 1, and 2 ng g^−1^ and measured at the beginning, middle, and end of each analysis sequence to correct for potential signal drift. Blank corrections were performed by analyzing ultrapure water before each sample and subtracting the resulting signal from that of the samples. The ultrapure water used for blank analysis was stored in the same precleaned polypropylene vials as the ice core samples. A total of 2090 EGRIP samples was analyzed following the bag-mean resolution, covering a range from 14 to 1254 m. Samples between 1049 and 1089 m were excluded due to data quality issues. To assess the repeatability of the measurements, 530 samples were reanalyzed, yielding a percentage difference of ~8% compared to the original results.

### EGRIP accumulation flux calculation

The EGRIP Hg accumulation flux is calculated by the following equation

Hg flux=[Hg]×snow accumulation rate

where [Hg] is the measured bag-mean concentration of total Hg and the snow accumulation rate is the modeled upstream past accumulation rate (*31*). Snow accumulation rates at the Northeast Greenland Ice Stream drilling site varied throughout the Holocene. The lowest snow accumulation rate was found during the transition from the Last Glacial Period into the Holocene, and it gradually increased to the highest level during the mid-Holocene (Fig. 2C). After that, the snow accumulation rate slowly decreased during the late Holocene (Fig. 2C).

The EGRIP Br flux was calculated in a similar way by multiplying the measured bag-mean concentration of Br and the modeled upstream past accumulation rate.

### Change point analysis

To identify abrupt changes in the Hg concentration and flux, a change point analysis was conducted in MATLAB with the *findchangepts* function. To avoid large data gaps during the early and mid-Holocene, continuous measurements of natural log-transformed Hg concentration and flux during 34 to 4500 years b2k were selected. The analysis aimed to find abrupt changes in the mean of respective sections before and after each point; the minimal number of samples in one section was set at 30, representing a period of 120 to 180 years; the minimum threshold for detection was set as three times the SD of the analyzed dataset.

### Box model simulation

To assess the role of Br concentration changes and temperature variations on the Hg deposition at EGRIP, a box model based on current-day atmospheric Hg chemistry was used. We assume all depositional and postdepositional processes remain similar during the Holocene because the climate excursions are relatively mild with no major changes in snow accumulation rates, dust deposition, and sea ice conditions (Fig. 2). This box model is based on the chemistry-climate model WACCM (*89*), including 86 chemical species (30 of which are Hg species), 290 thermal reaction rates, and 75 photolysis rates. Photolysis and heterogeneous reaction rates are extracted from a global WACCM simulation, whereas gas phase thermal reaction rates are calculated online on the basis of temperature and pressure. The deposition of oxidized mercury [Hg(II)] species is assumed to be the relevant output parameter correlated with the measured EGRIP Hg fluxes, and the deposition lifetime of Hg(II) species is set to the WACCM tropospheric average (19 days). The simulations were run for lower troposphere conditions (surface up to 500 hPa) for the atmospheric composition relevant to the 4.2-ka event period to evaluate the impact of increased atmospheric Br concentrations and higher temperatures on Hg fluxes.

The box model simulations were conducted under “low-Br” and “high-Br + 5 K” conditions, where “high-Br + 5 K” was assumed to be 88% larger in Br concentrations and 5 K warmer than “low-Br” in accordance with the changing environmental proxies observed during the 4.2-ka event (Fig. 2). The box model conditions aim to represent the global atmosphere at 4.2 ka b2k. The following concentrations of atmospheric gases were used: CH_4_ at 641 parts per billion (ppb) (*90*), CO_2_ at 275 parts per million (ppm) (*91*), N_2_O at 270 ppb (*92*), and Hg(0) at 0.27 ng m^−3^ (*36*). Given the lack of data for other trace gases for this time period, we conducted 1000 sensitivity simulations varying the value of trace gas concentrations by a factor of 2 around a central value. The central values were selected as follows: 100 ppb for CO (*93*), 3 parts per thousand (ppt) for Br_y_ (total inorganic Br: Br + BrO + HBr + HOBr + BrCl + BrNO_2_ + BrNO_3_) under low-Br conditions (high-Br conditions are multiplied by 1.88), 10 ppt for Cl_y_, 0.7 ppt I_y_, 4 ppt for NO, 8 ppt for NO_2_, and 10 ppb for O_3_. A set of 1000 parameter values for these concentrations were selected with the Latin Hypercube Sampling (*94*), which enables simulations distributed evenly throughout the uncertainty space of atmospheric composition for the 4.2-ka period. For each of these 1000 simulations, we evaluate the ratio of the Hg(II) deposition flux in the “high-Br + 5 K” to “low-Br” cases. The full distribution of results is shown in fig. S6, with the spread in outcomes representing the uncertainty in atmospheric composition for the time period. Nevertheless, the calculated impact of a global Br increase and a warmer temperature on Hg fluxes (24 to 92%; median: 53%) is smaller than the measured Hg flux increase during this time period (214%), suggesting that increased Br oxidation is not the major factor driving the increasing Hg fluxes. It must be noted that our modeling approach assumes a global change in Br concentrations and analyzes the global difference in Hg(II) deposition, not considering impacts of spatially distributed changes, which are difficult to assess given the lack of available data for this time period.
