# Supplementary material for: Mercury accumulation over the Holocene revealed from a Greenlandic ice core
Source: Sci Adv. 2026 May 15;12(20):eaea0517. doi: 10.1126/sciadv.aea0517 (PMC13178554; doi:10.1126/sciadv.aea0517)
Supplement: Supplementary file 1 — Supplementary Text S1 and S2 Figs. S1 to S6 Tables S1 and S2 [file sciadv.aea0517_sm.pdf]

Science Advances
AAAS

Supplementary Materials for

Mercury accumulation over the Holocene revealed from a Greenlandic ice core

Zhiyuan Gao *et al.*

Corresponding author: Zhiyuan Gao, zhygcoe@hotmail.com; Feiyue Wang, feiyue.wang@umanitoba.ca

Sci. Adv. **12**, eaea0517 (2026)
DOI: 10.1126/sciadv.aea0517

This PDF file includes:

Supplementary Text S1 and S2
Figs. S1 to S6
Tables S1 and S2

Text S1. Comparison between EGRIP and other published ice core Hg records

Over the recent millennium, the Hg concentration range and temporal trends of the EGRIP record generally agree with those reported in previous studies using ice cores from the Arctic, North America and Asia (Table. S1 and Fig. S3). Since the industrial period around ~1850, all records showed a prominent increase. However, identifying a pre-industrial Hg signal and defining a natural baseline is less straightforward in previously published ice core records, which only extended back to the 14th century.

The only attempt to characterize a period of natural variation was conducted by Beal et al. (2015) (*21*) using the Mt. Logan ice core record. The authors selected pre-1570 CE levels to represent natural deposition and variations, and identified a modest Hg increase between ~1600 to 1850 (*21*). On the other hand, Eyrikh et al. (2017) (*22*) questioned whether the base of the Belukha glacier ice core samples (1680 CE – 1810 CE) had already been influenced by anthropogenic emissions, as the oldest sample stopped on a decreasing trend (Fig. S3E).

Overall, properly identifying a natural baseline level is critical for identifying pre-industrial Hg signals. Since 254 CE (Change point at 1746 yr b2k, Table. S1), the EGRIP record showed periods of accelerating Hg accumulation, and this time point is selected in our study to define the natural baseline. Notably, none of the previously published ice core studies have covered this period.

Text S2. Long-term variations of ice core proxies during the late Holocene

During the late Holocene, a sustained, slow increase in the Hg concentration and ice core Hg accumulation flux was identified by the trend analysis (Fig. S4) and histogram distribution (Fig. S5), respectively. This long-term Hg increase could be partially attributed to the growing contribution from human activity, as discussed in the Main Text, and potentially to variations in ice core proxies shown in Fig. 2. To further evaluate influences from long-term trends in snow accumulation rates, Br flux, Ca concentrations, a change point detection and trend analysis was carried out for each proxy using the same approach described for Hg in the Materials and Methods section (Table. S2 and Fig. S4).

For ice accumulation rates during the past 5,000 years, only one change point was identified at 3958 yr b2k, corresponding to a decreasing transition. This suggests a period of lower ice accumulation rates over the past ~4 millennia. Trend analysis further confirmed that snow accumulation rates have been slowly decreasing, except for a short interval around 2.0 ka b2k (Fig. S4). The decreasing snow accumulation could have caused extended air-snow exchange, potentially leading to enrichment of atmospheric Hg deposition in snow and contributing to increased ice Hg accumulation. In the meantime, it could have also extended surface Hg photochemical re-emission processes, the net influence of which remained largely uncharacterized under paleoclimate conditions. Furthermore, during the early Holocene and the 4.2 ka event, increases in Hg accumulation were accompanied by increasing snow accumulation and variations in other proxies. Thus, the net effect of changing ice accumulation rates on ice Hg flux remains unresolved at this time.

Analysis of the Br trend suggests that the Hg flux is more closely linked with processes influencing Hg concentrations (e.g., atmospheric Hg deposition and Hg re-emission processes)

and rather than the snow accumulation process. During the late Holocene, multiple oscillations in the Br flux were delineated by change point detections (Table. S2), but only the increase during 4110–3917 yr b2k coincided with the 4.2 ka Hg flux increase, which has been addressed in the Main Text. Notably, the trend analysis of Br flux showed a similar pattern to that of snow accumulation over the entire Holocene (Fig. S4). In other words, a faster decrease in snow accumulation rates was generally accompanied by a faster decrease or a slower increase in Br flux, whereas this pattern was not consistently observed between snow accumulation rates and Hg flux. Throughout the Holocene, the Hg flux was weakly correlated with snow accumulation rate ($r = -0.11$, $p < 0.01$), whereas a much stronger correlation was found between the Br flux and the snow accumulation rate ($r = 0.67$, $p < 0.01$). In comparison, the Hg flux was strongly correlated with the ice core Hg concentration ($r = 0.99$, $p < 0.01$), while the Br flux showed a weaker correlation with the Br concentration ($r = 0.64$, $p < 0.01$).

For Ca concentrations during the late Holocene, statistical analysis showed multiple oscillations without a long-term trend consistent with that of Hg variations (Table. S2 and Fig. S4). The magnitude of these Ca oscillations during the late Holocene was substantially lower than that during the transition from the Last Glacial Period into the Holocene when dust-bound Hg contributed relatively a minor role (*25*). Therefore, oscillations at low Ca levels are unlikely to play a major role in delivering dust-bound Hg to drive the sustained millennial-scale Hg increase during the late Holocene.

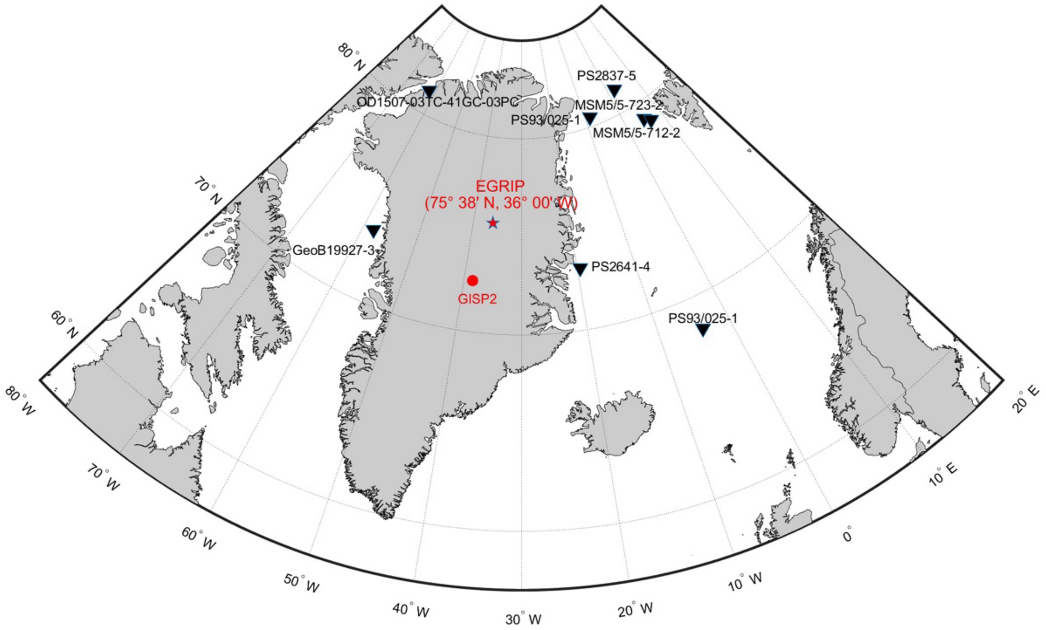

Figure S1. The location of the East Greenland Ice-core Project (EGRIP) ice coring site, identified by the red star. The red dot represents the location of the GISP2 core from which the calcium data are shown in Fig. 2 The dark triangles represent the locations of sediment cores that provided the sea ice biomarker (P_BIP_{25}) data shown in Fig. 2.

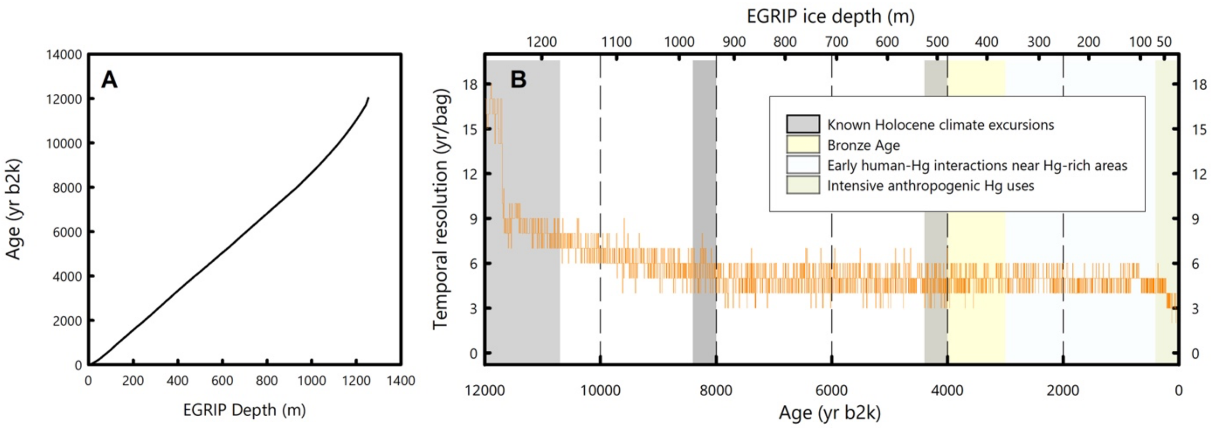

Figure S2. The age-depth profile (A) and temporal resolution (B) of the EGRIP ice core samples over the Holocene. For the most recent sample (34 yr b2k) to 8.0 ka b2k, there are minor variations in the temporal resolution; for those older than 8.0 ka b2k, the temporal resolution decreases.

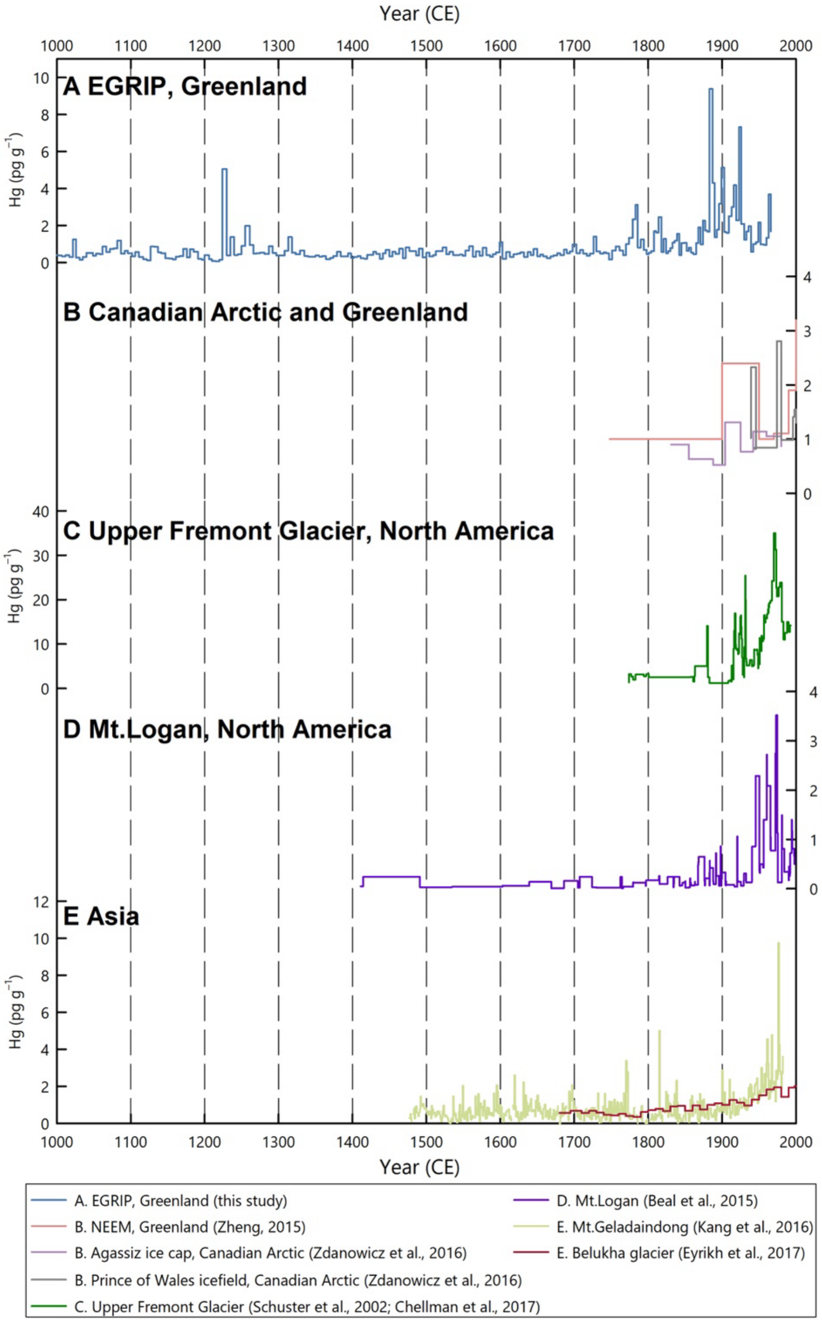

Figure. S3 Comparisons of the ice core Hg concentration between EGRIP and other published ice core records from the Arctic, North America and Asia. Panel A shows data at the multi-annual resolution from this study; panel B shows data at the multi-decadal resolution from the NEEM ice core (*31*), Agassiz ice cap (*32*) and Prince of Wales icefield (*32*); panel C shows data at the multi-annual resolution from the Upper Fremont Glacier (*29, 30*); panel D shows data at the multi-annual resolution from Mt. Logan (*21*); and panel E shows data at the multi-annual resolution from Mt. Geladaindong (*23*) and data at the decadal resolution from the Belukha glacier (*22*).

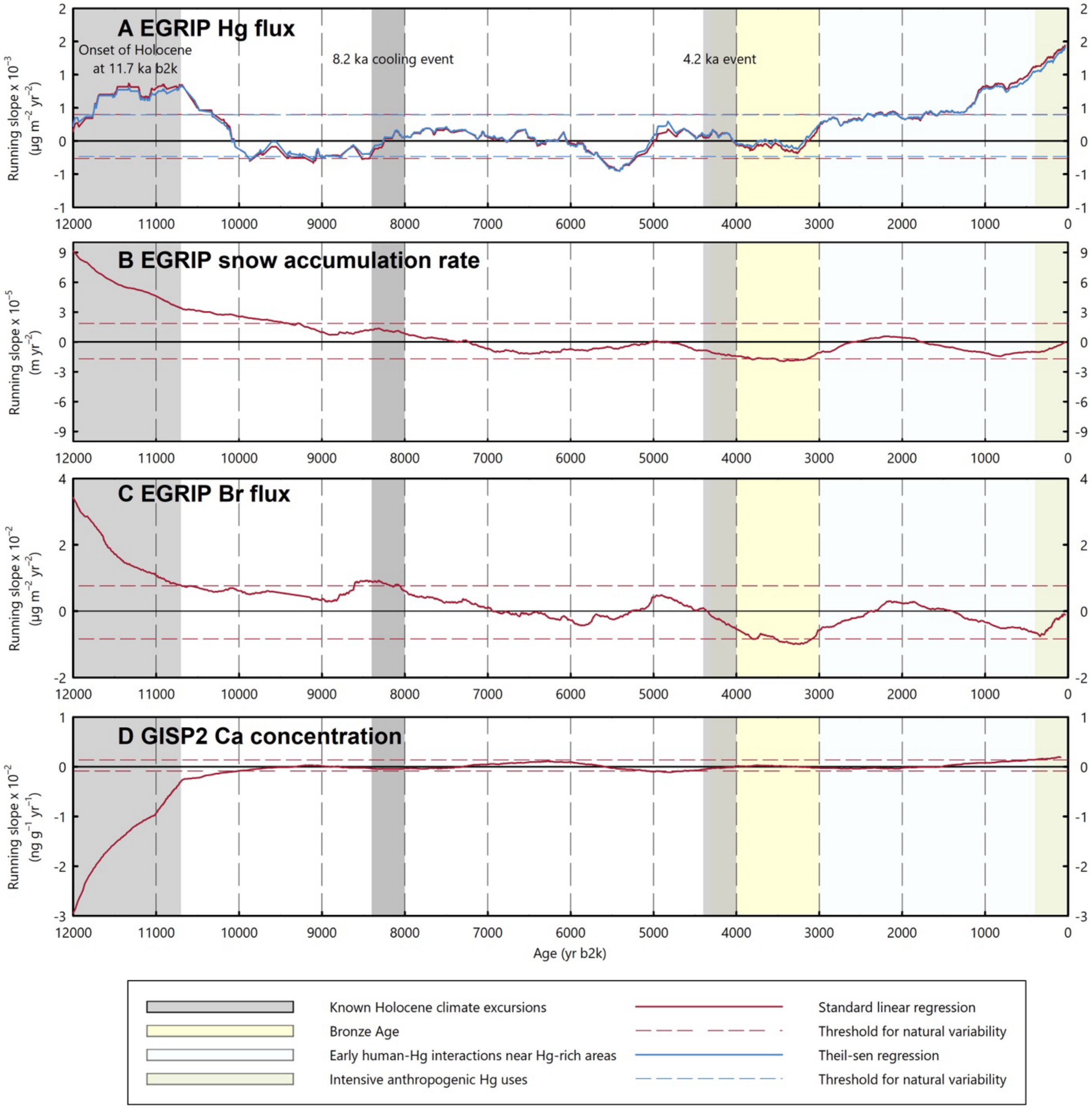

Figure S4. Trend analysis for Hg and ice core proxies during the Holocene. Shown in the figure are the millennial-scale running slopes of the standard linear regression (in red) and Theil-Sen regression (in blue) of (A) log-transformed Hg flux, (B) snow accumulation rates, (C) EGRIP Br flux, and (D) GISP2 Ca concentration. The thresholds for natural Holocene variability are determined as the 95th quantile (upper limit) and the 5th quantile (lower limit) of the running slope values between 10,000 – 1,746 yr b2k for log-transformed Hg flux and between 10,000 – 34 yr b2k for other parameters. In panel A, a value above the upper threshold means that the Hg flux is increasing faster than Holocene variability.

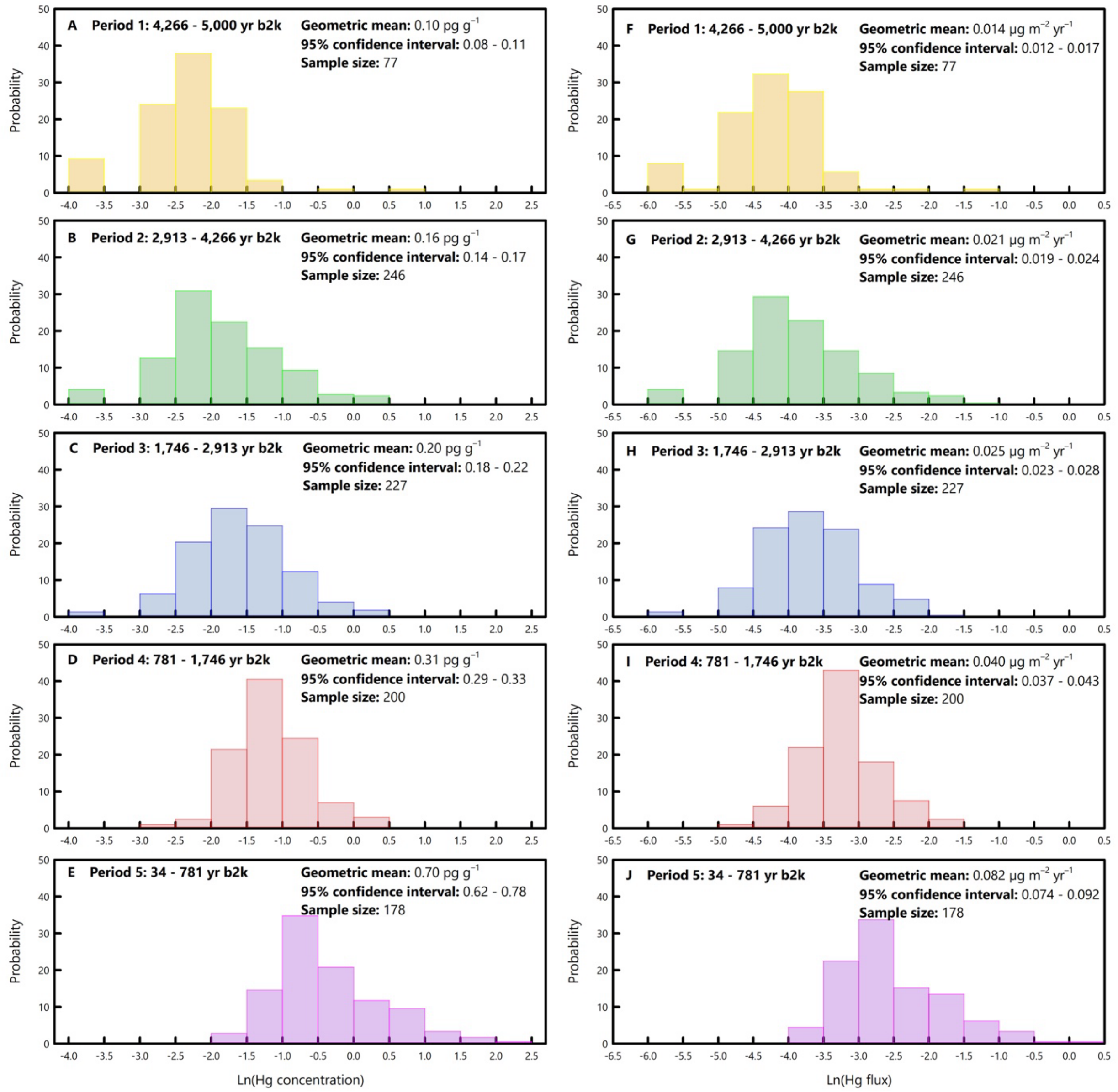

Figure S5. Comparisons of the distribution of log-transformed Hg concentrations (left) and fluxes (right) in the five periods over the recent 5,000 years. The periods are delineated by change-point detections. The geometric mean, 95% confidence interval and sample size are provided for each period.

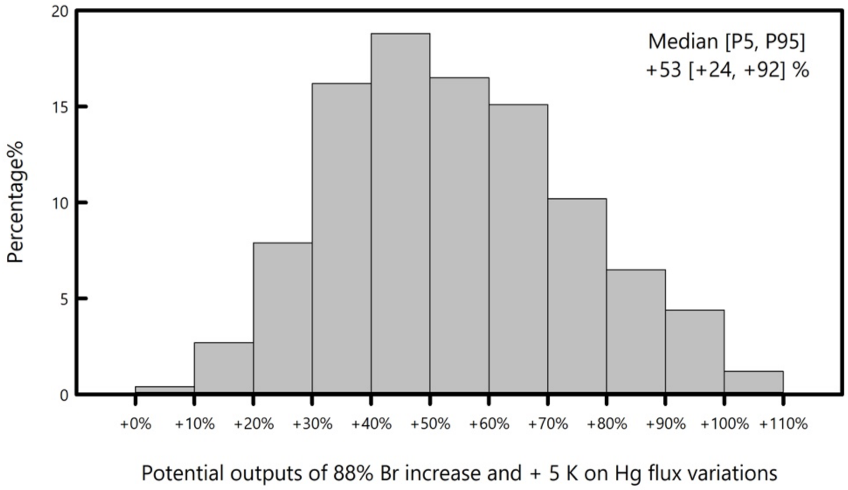

Figure S6. Histogram of the box model outputs on Hg flux variations as a result of + 88% increase in the Br concentration and + 5 K increase in temperature. The figure shows results of 1000 simulations. The median of the outputs (53%) indicates that the changing environmental conditions can lead to ~53% increase in the Hg flux.

Table S1. Summary of the concentration range and temporal coverage for ice core Hg studies for the time period since 1000 CE

Location	Concentration range ($pg\ g^{-1}$)	Temporal coverage	Source
EGRIP, Greenland	0.07 to 9.4	1000 – 1966 CE	This study
NEEM, Greenland	1.0 to 3.2	1978 – 2010 CE	(*31*)
Agassiz ice cap, Canadian Arctic	0.44 to 2.4	1808 – 1980 CE	(*32*)
Prince of Wales icefield, Canadian Arctic	0.17 to 2.8	1918 – 2004 CE	(*32*)
Upper Fremont Glacier, North America	1.2 to 35.0	1740 – 1998 CE	(*29, 30*)
Mt. Logan, North America	0.01 to 3.5	1411 – 1998 CE	(*21*)
Mt. Geladaindong, Asia	0.02 to 9.8	1477 – 1982 CE	(*23*)
Belukha glacier, Asia	0.07 to 8.9	1680 – 2001 CE	(*22*)

Table S2. Identified change points for Hg and ice core proxies during the late Holocene

Tested Parameter	Temporal resolution (yr)	Change points during an increasing transition (yr b2k)	Change points during a decreasing transition (yr b2k)
EGRIP Hg concentration and flux (log-transformed)	4 – 6	126, 235, 1746, 2913, 3490, 4266	1882, 3081, 3949
EGRIP snow accumulation rates	~10		3958
EGRIP Br flux	4 – 6	129, 666, 1342, 1813, 2239, 2695, 3005, 4110, 4473	420, 837, 1159, 1579, 2098, 2502, 2863, 3193, 3393, 3579, 3780, 3971, 4701, 4841
GISP2 Ca concentrations	~5	569, 3040, 3765, 4077, 4852	786, 2762, 3344, 3924, 4577
